# Importance of LINC00852/miR-145-5p in breast cancer: a bioinformatics and experimental study

**DOI:** 10.1007/s12672-024-01553-5

**Published:** 2024-11-18

**Authors:** Abbas Shakoori, Asghar Hosseinzadeh, Nahid Nafisi, Ramesh Omranipour, Leyla Sahebi, Nazanin Hosseinkhan, Mohsen Ahmadi, Soudeh Ghafouri-Fard, Maryam Abtin

**Affiliations:** 1 Department of Medical Genetics, School of Medicine, Tehran University of Medical Sciences, Tehran, Iran; 2 Department of Medical Genetics, Cancer Institute of Iran, Imam Khomeini Hospital Complex, Tehran University of Medical Sciences, Dr. Qarib St., Keshavarz Blvd, Tehran, Iran; 3 Department of Biology Education, Farhangian University, Tehran, Iran; 4 Surgery Department, Rasoul Akram Hospital, Clinical Research Development Center (RCRDC), Iran University of Medical Sciences, Tehran, Iran; 5 Breast Disease Research Center (BDRC), Tehran University of Medical Sciences, Tehran, Iran; 6 Department of Surgical Oncology, Cancer Institute, Tehran University of Medical Sciences, Tehran, Iran; 7 Maternal, Fetal and Neonatal Research Center, Family Health Research Institute, Tehran University of Medical Sciences, Tehran, Iran; 8 Endocrine Research Center, Institute of Endocrinology and Metabolism, Iran University of Medical Sciences (IUMS), Tehran, Iran; 9 Department of Medical Genetics, Shahid Beheshti University of Medical Sciences, Tehran, Iran

**Keywords:** LINC00852, miR-145-5p, Breast cancer

## Abstract

**Purpose:**

We aimed to examine the importance of an lncRNA, namely LINC00852, in the pathogenesis of breast cancer.

**Materials and methods:**

In the current study, we used several online tools to examine the importance of LINC00852 in breast cancer. Then, we examined these findings in 50 pairs of breast cancer tissues and adjacent non-cancerous ones. We also re-evaluated the data of miR-145-5p signature from our recent study.

**Results:**

While in silico tools revealed down-regulation of LINC00852 in breast cancer samples, expression assays showed significant up-regulation of this lncRNAs in breast cancer samples compared with matching control samples from Iranian patients. miR-145-5p was under-expressed in breast cancer samples compared with non-cancerous samples. LINC00852 could separate breast cancer tissues from adjacent non-malignant tissues with an AUC value of 0.7218 (P value < 0.001).

**Conclusion:**

The current study potentiates LINC00852/miR-145-5p axis as a possible contributor to the pathogenesis of breast cancer.

## Introduction

Breast cancer is the most frequent malignancy that impends female health with an approximate rate of 1.5 million newly diagnosed cases each year [1, 2]. The most effective way to overcome the cancer is prompt detection and precision treatment, which relies on identification of the pathogenesis of this malignancy. As the tumorigenic mechanisms of breast cancer are complex, discovery of novel biomolecular markers and therapeutic targets is crucial for lengthening patients' survival and refining their quality of life [3].

Long noncoding RNAs (lncRNAs) are a great category of transcripts which includes those with transcript length of over 200 nt with restricted protein coding potential [2, 4]. Recently, lncRNAs have attracted more attention to their emerging role as tumor biomarkers for early discovery, diagnosis, prognostic evaluations, and extrapolation of therapeutic response because of their functions in different cellular processes such as proliferation, apoptotic pathways, metastases, metabolism, chemoresistance, and stemness conservation [5, 6]. Up to now, several studies have mentioned lncRNAs as key regulator of gene expression at multiple levels, thus aberrant expression of lncRNAs is linked with the pathological reaction including tumor initiation and development [7, 8]. One of important role of lncRNAs is their action as competing endogenous RNAs (ceRNAs) to de‐suppress gene expression through competition with miRNAs for interface with common mRNA targets [9, 10]. Expression deregulation of lncRNAs that frequently was seen in breast cancer and other malignancies affects this miRNA/lncRNA interaction and can trigger tumorigenesis [11, 12]. Kong et al. demonstrated that lncRNA-CDC6 has a ceRNA function to decrease level of miR-215 as a molecular “sponge”, then up-regulating level its target gene CDC6, and promoting breast cancer proliferation and metastases [13]. Moreover, others demonstrated that SNHG14 lncRNA has a ceRNA function and stimulates proliferation of breast cancer cells via adsorbing miR-543 and further regulating level of KFL7 [14].

Single-cell RNA-sequencing (scRNA-seq) studies have revealed the extent of intratumoral heterogeneity in lncRNA expression in various cancers, including breast cancer [15–17]. By applying these technologies, it becomes possible to map the distribution of long intergenic non-protein coding RNA 852 (LINC00852) across different cellular subpopulations, including cancer stem cells, immune cells, and tumor epithelial cells. Understanding this heterogeneity can provide insights into how LINC00852 may contribute to the varying response to treatment or disease progression. LINC00852 is a novel lncRNA being initially discovered in lung cancer [18]. Overexpression of LINC00852 was observed in ovarian cancer [19], hepatic cancer [20], prostate cancer [21], stomach cancer [22], osteosarcoma [23] and colon carcinoma [24] in relation with proliferation, metastasis, invasion and chemoresistance. Yet, function of LINC00852 in breast cancer is not comprehensively clear.

LINC00852 has been shown to act as ceRNA for miR-145-5p in lung cancer [25]. miR-145-5p has been verified as a tumor suppressor in diverse malignancies, including breast cancer [26–28]. According to the majority of studies, miR-145-5p is one of the main tumor suppressors in the cells that is downregulated in breast tumor tissue and related with tumor promotion, invasion, drug resistance and poor prognosis of patients [29–31].

In the present study, we used several online tools to examine the importance of LINC00852 in the pathoetiology of breast cancer. We also measured the expression of LINC00852 in the tumor and non-tumor tissues of breast cancer patients. Furthermore, we re-analyzed expression pattern of miR-145-5p from our previous study [32]. These examinations are expected to enhance understanding of the common effect of this lncRNA/miRNA pair on the expression of miR-145-5p targets that are involved in cancer‐associated pathways.

## Materials and methods

### Assessment of LINC00852 expression

In order to gather information regarding the expression levels of LINC00852 in breast cancer, we made use of multiple databases. Initially, we conducted pan-cancer analysis to determine the expression levels of LINC00852 in breast cancer by extracting information from the GENT2 database [33], accessible at http://gent2.appex.kr/gent2/. Afterward, we employed the bc-GenExMiner v5.1 database, accessible at http://bcgenex.ico.unicancer.fr/BC-GEM/GEM-requete.php, to compare its expression in cancer tissues, tumor-adjacent tissues, and normal samples. This comparison was based on the TCGA and GTEx data [34]. To confirm the consistency of the obtained data, we further utilized the ENCORI/starBase (https://rnasysu.com/encori/index.php), to evaluate the expression of LINC00852 in both cancer and normal samples based on the TCGA data with different sample sizes [35]. Through these comprehensive analyses, we aimed to attain a complete understanding of the expression patterns of LINC00852 in breast cancer.

### Analysis of the correlation of LINC00852 expression with clinicopathological parameters

We collected data on the association between LINC00852 expression and various clinicopathological parameters, including stages, age, PAMS50, and tumor subtypes. To analyze this correlation, we utilized two databases: bc-GenExMiner v5.1 and UALCAN (https://ualcan.path.uab.edu/index.html).

### Identification of subcellular localization of LINC00852

To determine the potential subcellular localization of LINC00852, we employed the iLoc-LncRNA(2.0) database. This database, which can be accessed at https://lin-group.cn/server/iLoc-LncRNA(2.0)/, utilizes a binomial distribution approach to envisage the subcellular locations of lncRNAs [36–38].

### Analysis of prognostic significance of LINC00852

The connection between LINC00852 expression and overall survival (OS), progression-free survival (PFS), disease-free interval (DFI), and disease-specific survival (DSS) was investigated using the ENCORI/starBase and Kaplan–Meier Plotter (https://kmplot.com/analysis/) database [39] and GSCA (https://guolab.wchscu.cn/GSCA/#/) tool [37].

### Analysis of immune cell infiltration

The GSCA webtool was employed to assess the detailed information of the correlations between LINC00852 level and immune infiltration in breast cancer.

### Samples

Fifty pairs of cancer tissues and corresponding non-malignant adjacent tissues were collected from patients who had undergone tumor resection at two surgery centers, Tehran, Iran. Written informed consent was signed by all participants. Fresh tissues were gathered in RNA Later substance (Biobasic, Canada). The inclusion criteria were: confirmed diagnosis of breast cancer, availability of detailed clinical and histopathological data, and patients' willingness to participate. Exclusion criteria were preoperative chemo/radiotherapy and presence of other systemic disorders. The study was allowed by the Ethics Committee of Tehran University of Medical Sciences, Tehran, Iran (IR.TUMS.MEDICINE.REC.1401.272). All procedures were in accordance with the ethical standards of the institutional and national research committee and with the 1964 Helsinki declaration and its later amendments.

### RNA extraction and cDNA production

Total RNA was extracted using TRIzol (Invitrogen) in the way that the producer stated. Integrity and concentration of RNA were judged using gel electrophoresis and spectrophotometer (NanoDrop 2000, Thermo Scientific), respectively. Conversion of RNA to cDNA was accomplished by ExcelRT™ 1st Strand cDNA Synthesis Kit (SMOBIO). Temperature conditions for cDNA synthesis were as following: 5 min at 70 °C, 10 min at 25 °C, 50 min at 50 °C and then 5 min at 85 °C.

### Real-time PCR

Expression level of LINC00852 was detected using the RealQ Plus 2 × Master Mix Green no Rox (Ampliqon, Denmark). GAPDH was used as normalizer. The 2 − ΔΔCt method was used for comparisons. The primers are displayed in Table S1. PCR program consisted of 5 min initial denaturation at 95 °C, 30 cycles of denaturation at 95 °C for 5 s and annealing at 60 °C for 20 s.

### Statistical analysis

The GraphPad Prism 8.3.4 (San Diego, CA) was used. Expressions of LINC00852 were calculated and compared between tumors and adjacent samples using the paired sample t test. Correlation between expressions of LINC00852 and miR-145-5p was assessed using Spearman correlation coefficient. Associations between expression of LINC00852 and clinical characteristics were judged by Mann–Whitney and one-way ANOVA tests (Kruskal–Wallis). In addition, the GraphPad Prism v.8.3.4 software demonstrated the receiver operating characteristic (ROC) curves. P value < 0.05 was considered as significant.

## Results

### Analysis of LINC00852 expression

**Fig. 1:**
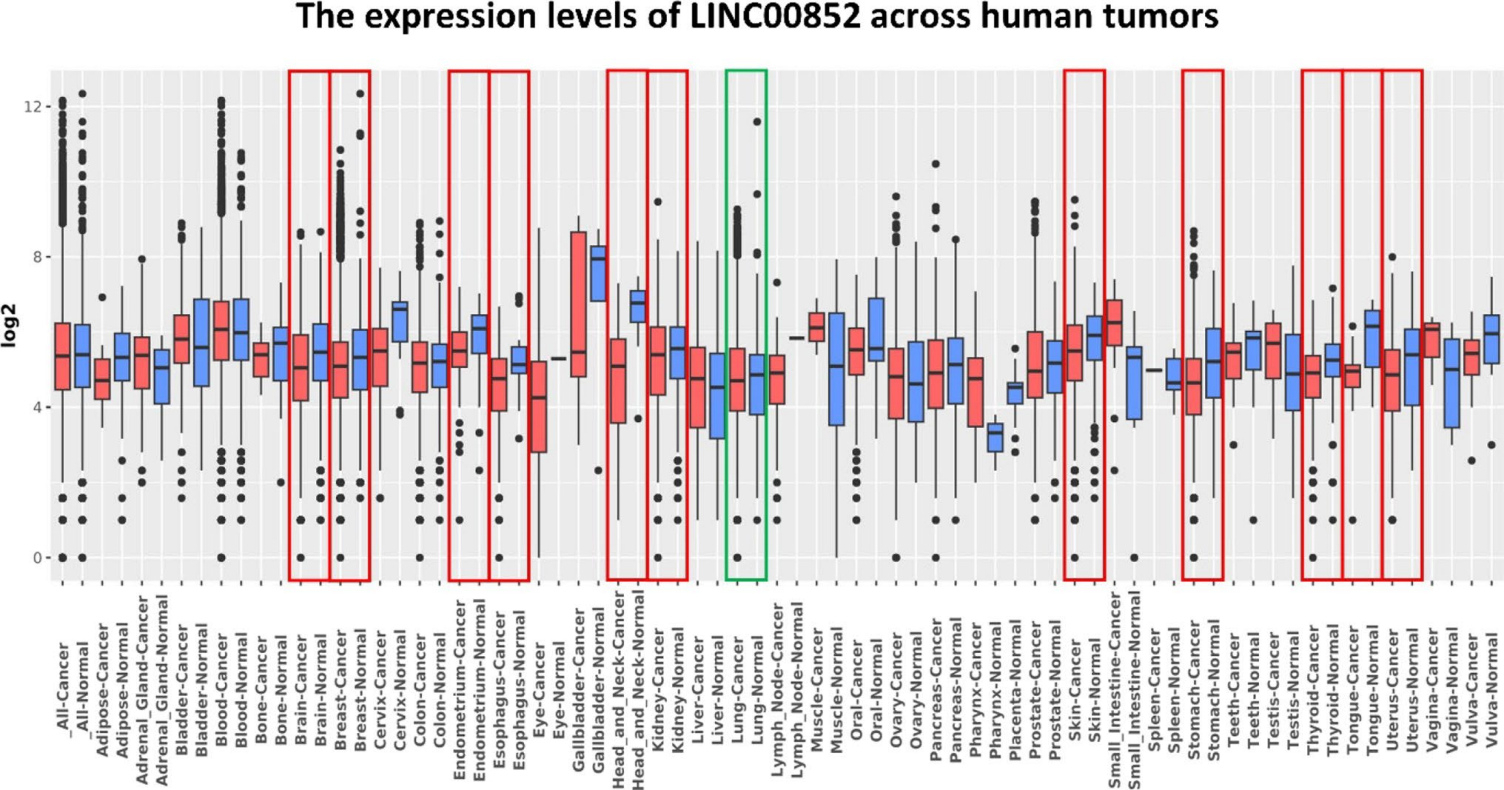
The expression pattern of LINC00852 across human cancer tissues. The data acquired from the GENT2 database. The red rectangles highlight cancers with significant decreases, while green rectangles denote those showing notable increases in expression of LINC00852

The pan-cancer analysis performed through the GENT2 database also demonstrated a significant upregulation of LINC00852 in lung cancer, and its remarkable downregulation in many tumors including brain, head and neck, esophagus, kidney, endometrium, stomach, tongue, thyroid, uterus and specially breast cancer (Fig. 1). Detailed statistics are shown in Table S2.

**Fig. 2:**
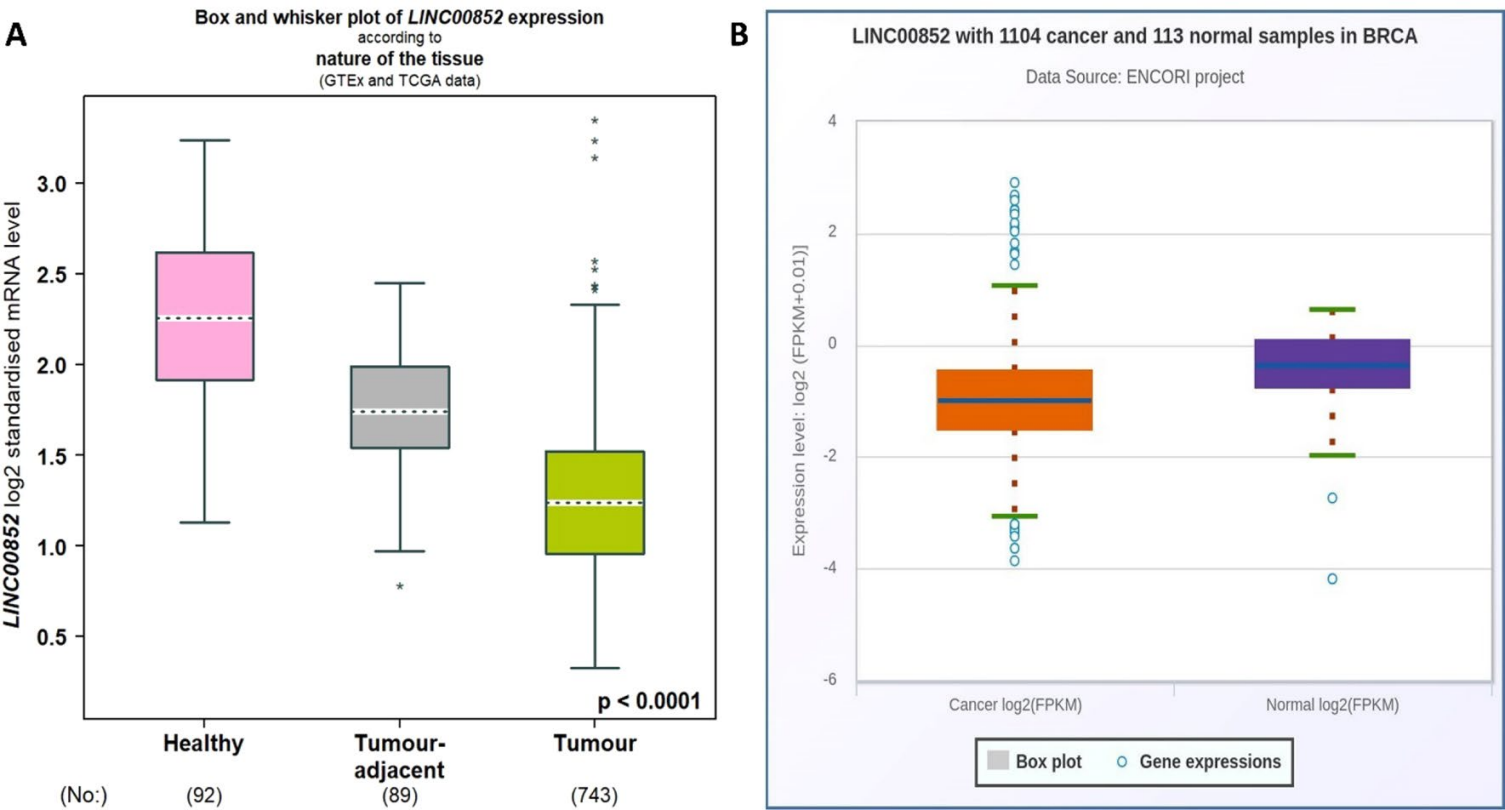
The expression pattern of LINC00852 in breast cancer. The expression data of LINC00852 in breast cancer tissues, tumor-adjacent tissues, and healthy tissues based on the TCGA and GTEx data, acquired from the bc-GenExMiner v5.1 database (Tumor-adjacent < Healthy: P < 0.0001, Tumor < Healthy: P < 0.0001, Tumor < Tumor-adjacent: P < 0.0001) **A**. The expression levels of LINC00852 in breast cancer tissues according to the TCGA data collected from ENCORI/starBase (Expression in tumor samples = 0.62, Expression in normal samples = 0.82, Fold change = 0.76, P value = 4.2e-11) (**B**)

Then, the expression levels of LINC00852 were found to be lower in both tumor tissues and adjacent tissues compared to healthy samples, as indicated by the bc-GenExMiner v5.1 database. Furthermore, the expression of LINC00852 was downregulated in tumor tissues when compared to tumor-adjacent tissues (Fig. 2A). Additionally, data obtained from the ENCORI/starBase database, as depicted in the Fig. 2B, revealed a significant reduction in LINC00852 expression in tumor tissues compared to healthy control samples. Detailed statistics are shown in Tables S3 and S4.

### Analysis of the correlation of LINC00852 expression with clinicopathological parameters.

**Fig. 3:**
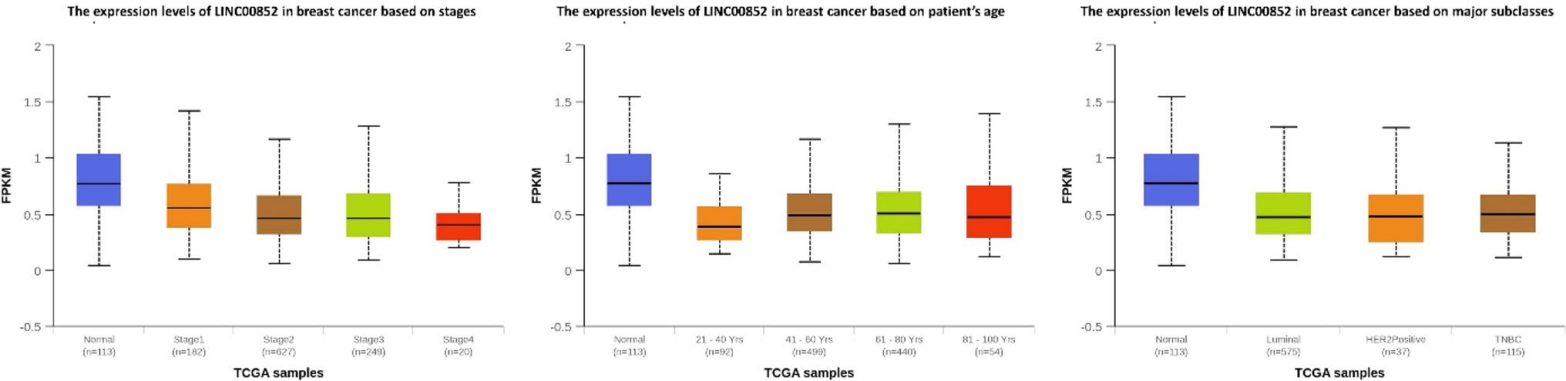
The correlation of LINC00852 expression with clinicopathological data in breast cancer. The expression of LINC00852 in breast cancer tissues based on cancer stage (**A**), patient’s age (**B**), and breast cancer major subclasses (**C**), obtained from the UALCAN database

The analysis conducted by UALCAN showed reduction in the levels of LINC00852 expression in breast cancer stages compared to normal samples. Specifically, the expression of LINC00852 was found to be higher in stage 2 compared to stage 4, but significantly lower in stages 3 and 4 compared to stage 1 (Fig. 3A). It was also observed that breast cancer patients between the ages of 41–60 and 61–80 had higher expression levels compared to those between the ages of 21–40 (Fig. 3B). In terms of major subclasses, the UALCAN database demonstrated that individuals identified as HER2 Positive, luminal, and TNBC had lower expression levels of LINC00852 compared to healthy individuals (Fig. 3C). Detailed statistics are shown in Table S5.

**Fig. 4:**
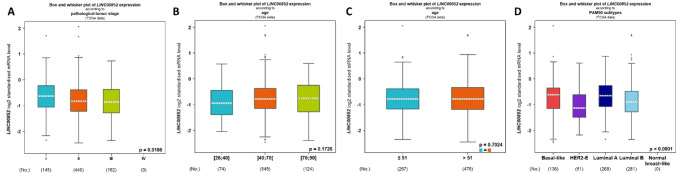
The correlation of LINC00852 expression with clinicopathological parameters in breast cancer. The LINC00852 expression data in breast cancer tissues according to cancer stage (**A**), patient’s age (**B**, **C**), and PAM50 subtypes (**D**), collected from the bc-GenExMiner v5.1

Furthermore, the bc-GenExMiner v5.1 data indicated a significant downregulation of LINC00852 expression in breast cancer stages 2 and 3 compared to stage 1 (Fig. 4A). When examining the expression data of LINC00852 based on age using the bc-GenExMiner v5.1 database, the database did not reveal any significant difference in LINC00852 expression between breast cancer patients aged ≤ 40, 40–70, and ≥ 70 years old (Fig. 4B), as well as between those aged ≤ 51 and > 51 years old (Fig. 4C). Additionally, the expression data based on PAM50 subtypes indicated that while the expression levels of LINC00852 were higher in Luminal A compared to HER2-E subtype, its expression was lower in Luminal B and HER2-E when compared to Luminal A and Basal-like subtypes, respectively (Fig. 4D). Detailed statistics are shown in Tables S6 and S7.

### Subcellular localization of LINC00852

Based on the data obtained from the iLoc-LncRNA(2.0) database, it was determined that LINC00852 is predominantly localized in the Cytoplasm, Cytosol.

### Analysis of prognostic significance of LINC00852

**Fig. 5:**
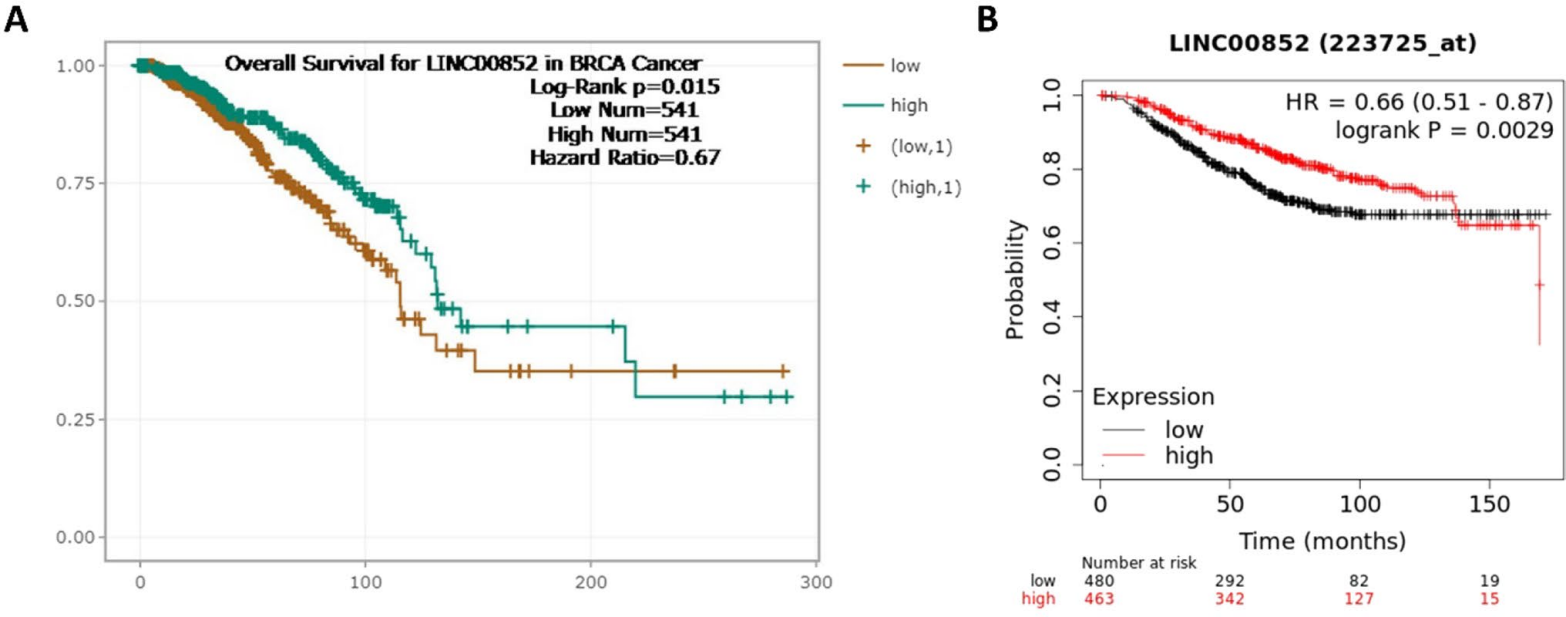
The association between the expression of the LINC00852 and the overall survival outcome of breast cancer in the TCGA database using the ENCORI/starBase (**A**), and Kaplan–Meier plotter (**B**)

Upon analyzing the OS of breast cancer patients, specifically focusing on the median expression of LINC00852, our investigation encompassed data obtained from various databases including ENCORI/starBase, Kaplan–Meier Plotter, and GSCA. As represented in Fig. 5, the findings from the ENCORI/starBase, Kaplan–Meier Plotter revealed that individuals with lower levels of LINC00852 in their tumor tissues exhibited a considerably higher risk of death compared to those with higher expression levels.

The GSCA database also showed that lower expression of LINC00852 was significantly correlated with poor OS. Nevertheless, it is worth noting that no statistically significant disparities were observed in terms of PFS, DFI, and DSS (Table 1).

**Table 1:** The survival difference of breast cancer patients between LINC00852 high and low gene expression groups

Survival outcome_type	High risk_categorical (H/L)	Log-rank p-value	Higher_risk_of_death
Overall survival (OS)	0.71509	0.03762*	Lower expression
Progression-free survival (PFS)	0.804688	0.118743	Lower expression
Disease-specific survival (DSS)	0.696492	0.096057	Lower expression
Disease-free interval (DFI)	0.958386	0.845921	Lower expression

^*^P-value < *0.05 considered as statically significant*

### Analysis of immune cell infiltration

To study the association between LINC00852 expression and the breast cancer tumor microenvironment, we conducted an evaluation of immune cell infiltration changes in samples exhibiting variations in LINC00852 expression. The data obtained from the GSCA database, as presented in Table 2, revealed correlation between the expression of LINC00852 in breast cancer and the infiltration of various immune cells. Notably, except for Central memory cells, Exhausted cells, B cells, CD4_naive cells, and Th1 cells, the infiltration level of other immune cells exhibited a significant relationship with the expression of LINC00852 in breast cancer. Infiltration of CD8_naive, Dendritic cells, Effector_memory, Macrophage, Monocyte, Neutrophil, Th17, Tr1, iTreg and nTreg were negatively correlated with expression of LINC00852. However, infiltration of other cells was positively correlated with expression of this lncRNA.

**Table 2:** The association between expression of the LINC00852 and infiltration of immune cells in breast cancer using the GSCA database

Cell_type	Correlation value	P-value
B-cell	0.044563	0.120083
CD4_T	0.382269	1.15E-43*
CD4_naive	0.043703	0.127415
CD8_T	0.185775	6.41E-11*
CD8_naive	-0.07511	0.008729*
Central_memory	0.055782	0.051618
Cytotoxic	0.177747	4.19E-10*
Dendritic Cell	-0.23544	8.37E-17*
Effector_memory	-0.12556	1.11E-05*
Exhausted	0.049312	0.085386
Gamma_delta	0.247862	1.65E-18*
MAIT	0.100906	0.00042*
Macrophage	-0.28696	1.61E-24*
Monocyte	-0.22903	5.83E-16*
NK	0.327572	7.4E-32*
NKT	0.153599	7.16E-08*
Neutrophil	-0.18883	3.07E-11*
Tfh	0.325141	2.19E-31*
Th1	0.028617	0.318322
Th17	-0.11559	5.27E-05*
Th2	0.105899	0.000214*
Tr1	-0.15995	1.99E-08*
iTreg	-0.05727	0.045687*
nTreg	-0.20466	5.52E-13*

^*^P-value < *0.05 considered as statically significant*

### Expression levels of LINC00852 in tumor tissues vs. non- tumor samples

**Fig. 6:**
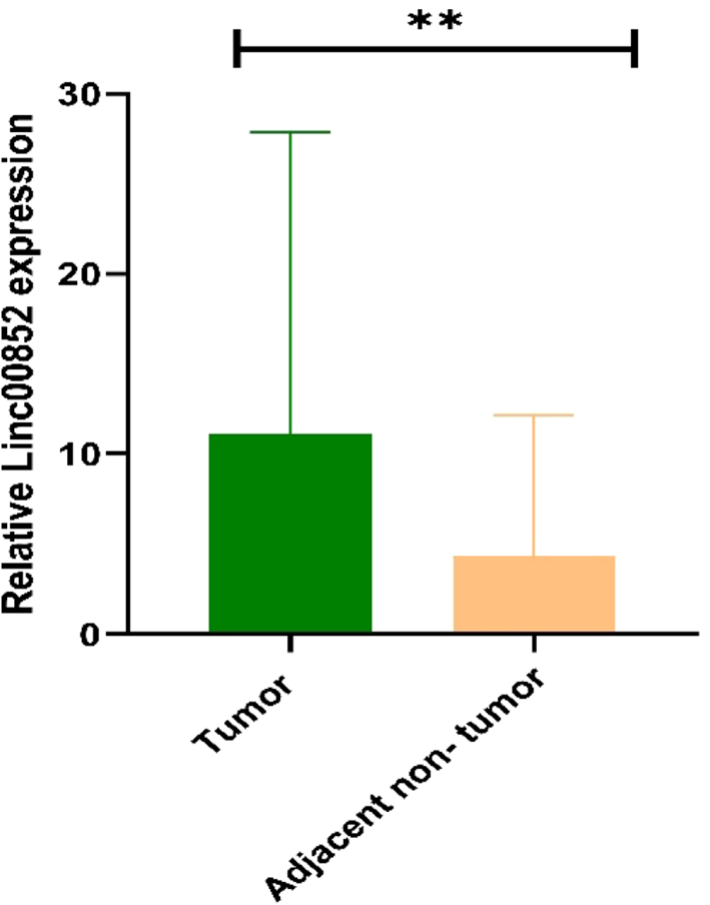
Relative expression of LINC00852 in breast cancer samples compared with adjacent control samples (P value < 0.01)

Contrary to the data from bioinformatics step, expression assays revealed significant up-regulation of LINC00852 in breast cancer samples compared with controls (Mean of relative expression in tumor: 10.14, mean of relative expression in non-tumor samples: 4.35, fold change (tumor versus non-tumor = 2.33) (Fig. 6).

Expression levels of LINC00852 were not correlated with any of clinicopathological features including age, tumor diameter, invasion to lymph, grade, stage, expression of hormone receptors, HER2 levels, KI67 levels or family history of this malignancy (Table 3).

**Table 3:** Correlations between clinical features of patients and LINC00852 expression (P: positive, N: negative, ER: estrogen receptor, PR: progesterone receptor)

	Subclass	Number of patients (%)	Median of LINC00852 level	IQR	Mean of LINC00852 level	SD	P value
Age	< 45	15	2.78	1.60	2.92	2.03	0.505
	45 or > 45	35	2.31	3.50	2.19	2.12	
Tumor diameter	< 2	7	3.68	2.90	3.14	1.55	0.24
	2–5	38	2.53	3.04	2.44	2.14	
	> 5	5	1.84	4.48	1.15	2.28	
Invasion to lymph	yes	29	2.96	2.11	2.94	1.98	0.07
	no	21	1.64	3.93	1.68	2.09	
Grade	1	13	2.31	3.35	2.34	1.87	0.88
	2	31	2.58	2.80	2.49	2.33	
	3	6	2.23	3.08	2.12	1.45	
Stage	1	11	3.28	2.41	2.93	1.46	0.51
	2	32	2.09	3.07	2.15	2.41	
	3	6	3.24	1.34	2.91	1.31	
	4	1					
ER	P	41	2.48	3.14	2.32	1.94	0.80
	N	8	3.28	3.62	2.86	3.03	
PR	P	38	2.31	3.14	2.31	1.93	0.693
	N	12	3.28	3.64	2.72	2.71	
HER2	P	43	1.64	4.22	1.93	2.04	0.56
	N	7	2.58	2.90	2.48	2.12	
KI67	< 16	19	2.31	3.07	2.45	2.28	0.84
	16 or > 16	31	2.58	2.20	2.38	2.02	
Family history	P	13	1.68	2.84	2.52	2.64	0.48
	N	37	2.78	2.34	2.37	1.92	

### Correlation analysis

**Fig. 7:**
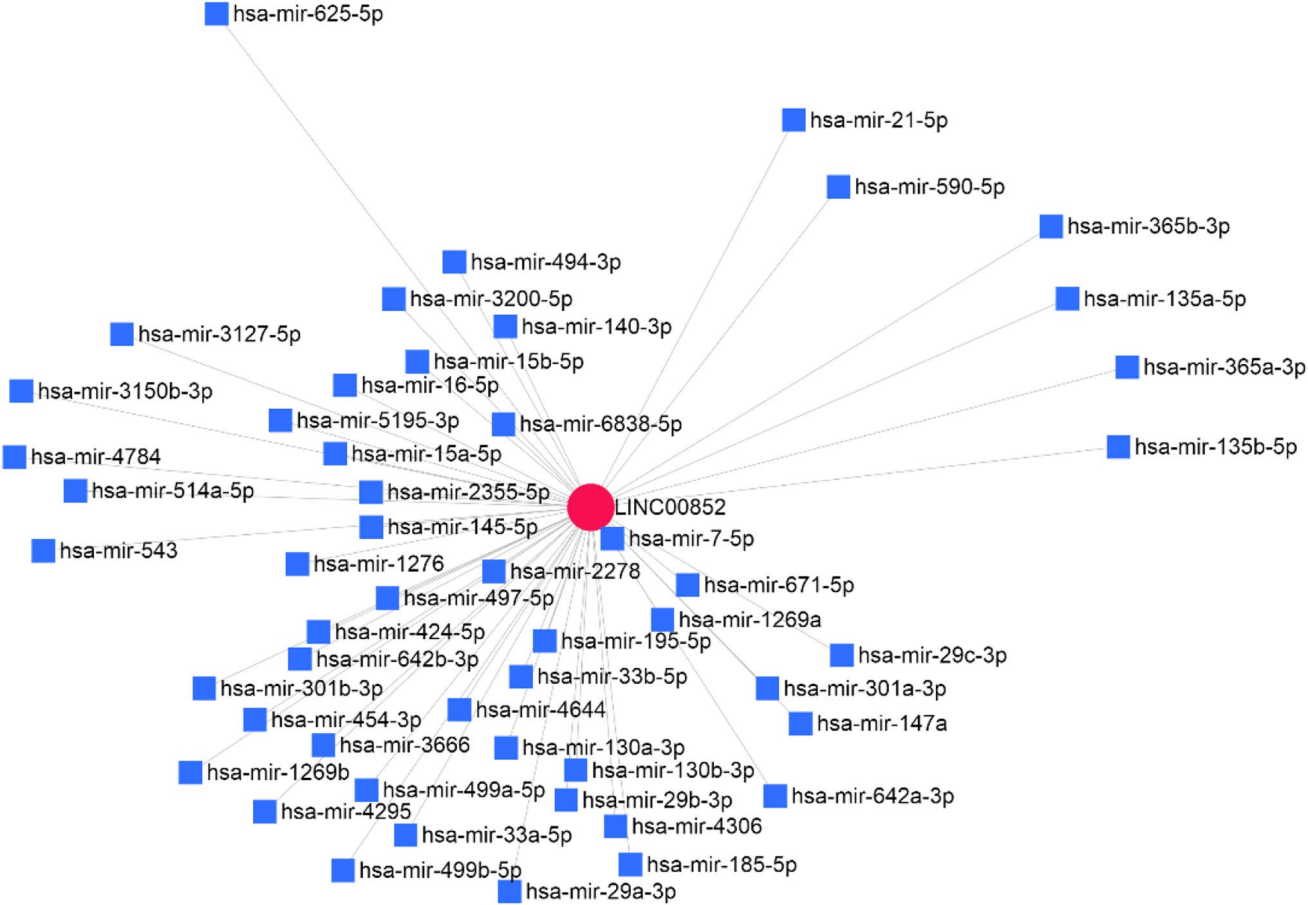
LINC00852 interaction with miRNAs such as miR-145-5p (www.mirnet.ca)

According to the target prediction using bioinformatics methods (Fig. 7, www.mirnet.ca), LINC00852 was predicted to be a ceRNA for miR-145-5p.

**Fig. 8:**
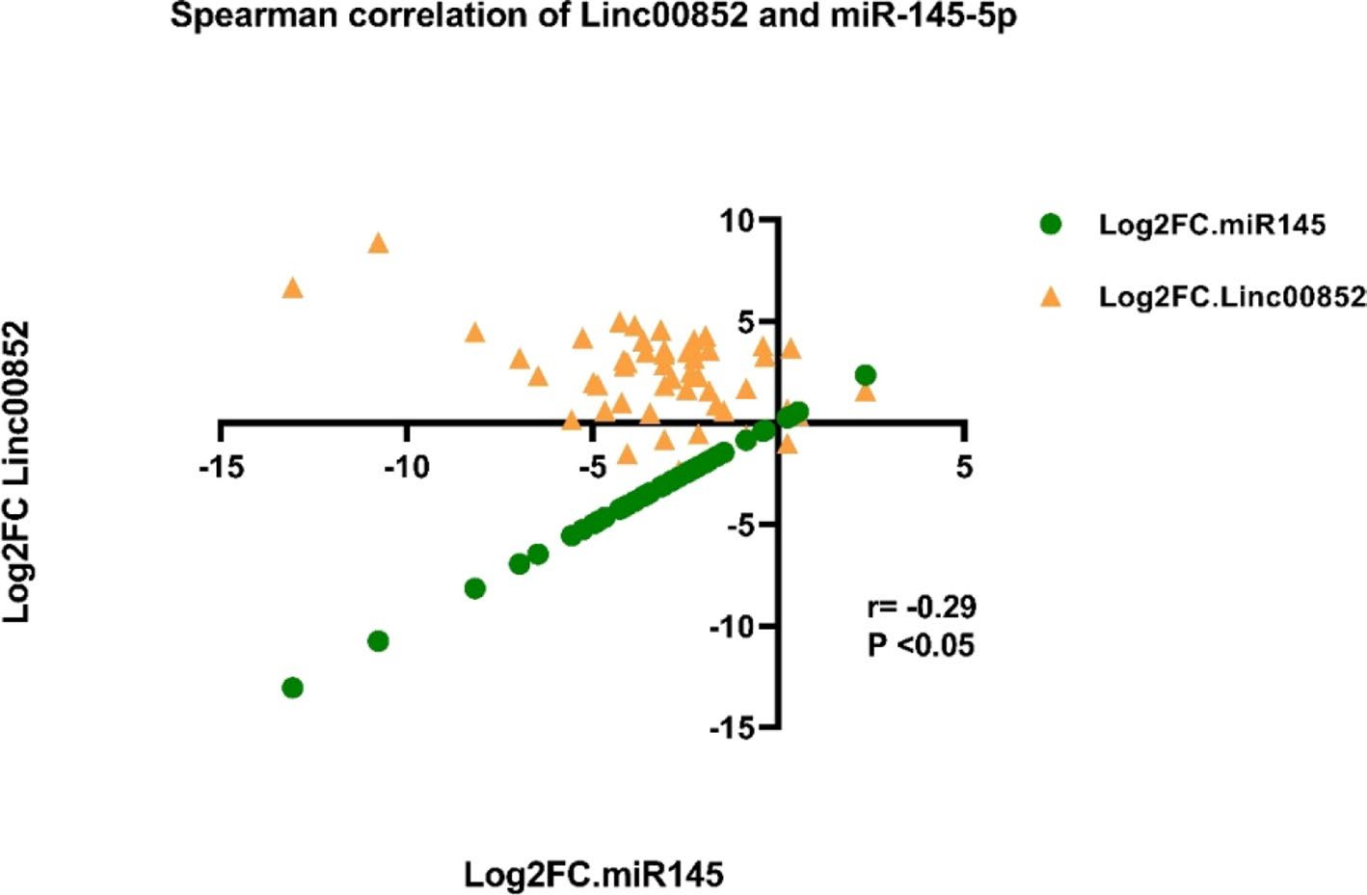
Correlation between LINC00852 and miR-145 expressions in breast tissues

Then, we evaluated the correlation between expression levels of LINC00852 and miR-145-5p using the log2FC values (Fig. 8). Expression levels of these transcripts were inversely correlated with r = -0.29 and P < 0.05.

### ROC curve analysis

**Fig. 9:**
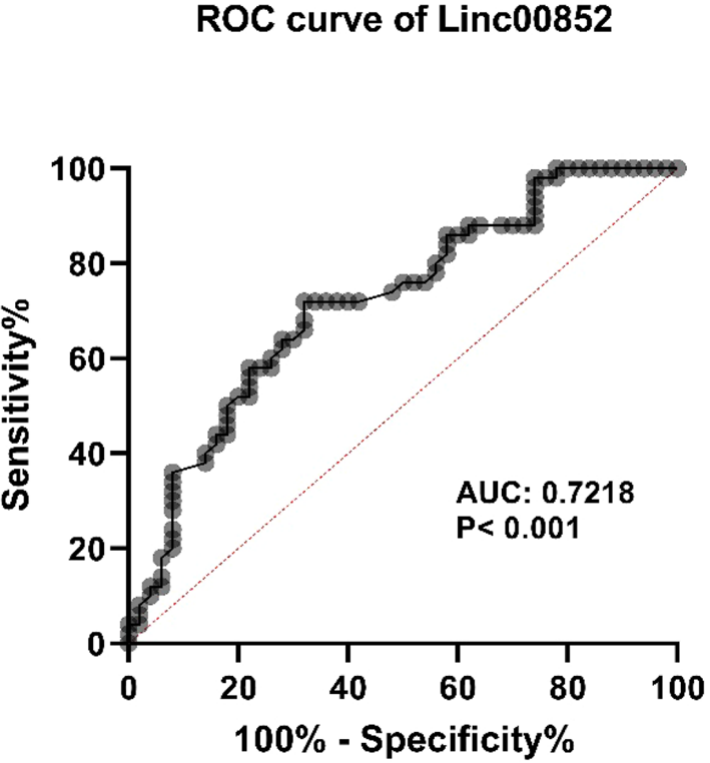
ROC curve of LINC00852 in breast cancer. Expression level of LINC00852 could differentiate between tumoral and non-tumoral samples with P value < 0.001)

The performance of LINC00852 as a diagnostic marker in breast tissues was examined using ROC curve which showed that LINC00852 could separate breast cancer tissues from adjacent non-cancerous tissues with an AUC value of 0.7218 (P value < 0.001, sensitivity: 76%, specificity: 70%) (Fig. 9).

## Discussion

Omics technologies have been extensively used in the field of breast cancer research and diagnostics to improve clinical outcomes [38]. In particular, single-cell omics offer deeper insights into cell-specific expression patterns, particularly in cancer stem cells [15]. LINC00852 has a dual function in the carcinogenesis. The pan-cancer analysis performed in the current study through the GENT2 database demonstrated a significant upregulation of LINC00852 in lung cancer, and its remarkable downregulation in many tumors including breast cancer. However, expression assays in the mentioned cohort of Iranian patients revealed its higher level in breast cancer tissues. The tumor suppressor miRNA, miR-145 was predicted to be sponged by LINC00852. Thus, the result of present study regarding down-regulation of LINC00852 in breast cancer is consistent with the proposed ceRNA network between these two transcripts. However, the inconsistency between the results of bioinformatics step and expression assays might be due to heterogeneity of breast cancer pathogenesis and the possible effects of ethnic or environmental/epigenetic factors on expression of this transcript.

In silico analyses revealed negative correlation between levels of LINC00852 and infiltration of CD8_naive, Dendritic cells, Effector_memory, Macrophage, Monocyte, Neutrophil, Th17, Tr1, iTreg and nTreg. However, infiltration of other cells was positively correlated with expression of this lncRNA. Previous studies in ER-positive breast tumors have indicated correlation between higher fraction of plasma cells and better disease-free survival (DFS), while greater numbers of M0 macrophages and Tregs have been correlated with lower OS [40]. Authors have also reported correlation between higher amounts of activated memory CD4 + T cells and better DFS in ER-negative or PR-negative subtypes [40]. Thus, the composition of tumor infiltrating immune cells has relation with prognosis in a subtype-dependent manner.

We could not detect correlation between clinicopathological data and expression of LINC00852 in our cohort of patients. This might be due to small sample size of the current study. However, in silico analyses showed higher expression of LINC00852 in stage 2 compared to stage 4, but significantly lower expression of this lncRNA in stages 3 and 4 compared to stage 1. Thus, this lncRNA has a stage-specific pattern of expression. This might imply specific role for this lncRNA in each stage of breast cancer. Moreover, lower expression levels of LINC00852 was detected in patients with HER2 positive, luminal, or TNBC subtypes compared to healthy individuals. In fact, almost all subtypes of breast cancer exhibited down-regulation of this lncRNA.

Implication of ceRNA network in the pathogenesis of breast cancer has been evaluated in several groups [41, 42]. Dysregulation of ceRNAs is strictly associated with the development and prognosis of malignancies, such as breast cancer [43]. The current study aimed at evaluation of a ceRNA axis, namely LINC00852/miR-145-5p in breast cancer. Contrary to the results of bioinformatics step, we detected over-expression of LINC00852 in breast cancer samples compared with non-cancerous tissues. Yet, we detected inverse correlation between levels of LINC00852 and miR-145-5p that supports the presence of the supposed LINC00852/miR-145-5p axis, as predicted by our initial in silico analyses. The functionality of LINC00852/miR-145-5p axis in the proliferation and chemoresistance has been verified in lung cancer [25]. It is worth mentioning that in addition to miR-145-5p, LINC00852 exerts its oncogenic effects through sponging several other miRNAs such as miR-140-3p [19], miR‐7‐5p [23] and miR-29a-3p [44]. Meanwhile, miR-145-5p can be sponged by some other lncRNAs such as DANCR [45] and PCAT1 [46]. These findings represent a complicated functional network between miRNAs and lncRNAs. Further studies are needed to find whether some sections of this network have specific functions in certain cancers.

Consistent with the tumor suppressor role of miR-145-5p, median of its expression was higher in patients with no invasion to lymph nodes. Besides, median of its expression was higher in HER2 positive samples [32].

Possible function of LINC00852/miR-145-5p axis as a diagnostic marker has been evaluated in the current study. In our previous study, we demonstrated that miR-145-5p had an appropriate performance in this regard [32]. Therefore, it is possible that inclusion of these transcripts in certain diagnostic panels of transcripts increases the sensitivity or specificity of these panels. It is worth mentioning that plasma level of miR-145-5p has been previously proved to precisely differentiate breast cancer patients from healthy persons [47].

The article provides a detailed analysis of LINC00852 as a potential biomarker in breast cancer, with an emphasis on its roles in tumorigenesis, metastasis, and chemoresistance. While databases like GENT2, bc-GenExMiner, and ENCORI were used for bulk tissue analyses, single-cell transcriptomics could offer deeper insights into cell-specific expression patterns, particularly in cancer stem cells.

In brief, LINC00852/miR-145-5p axis is a possible functional axis in the pathogenesis of breast cancer. The result of the current study should be confirmed in functional cell line studies as well as in larger sample sizes of clinical specimens of breast cancer.

## Limitation

Although our research has identified new diagnostic markers for breast cancer, it has some limitations. First, the results of the in silico step was not verified in the expression assays in clinical samples. Second, we did not perform in vitro and in vivo experiments to investigate the biological impact of LINC00852/miR-145-5p axis in breast cancer. Finally, the study lacks a focus on the heterogeneity of lncRNA expression at the single-cell level or across cancer subtypes.

## Data Availability

All data generated or analyzed during this study are included in this published article.
